# Hand Gesture Recognition Using Single Patchable Six-Axis Inertial Measurement Unit via Recurrent Neural Networks

**DOI:** 10.3390/s21041404

**Published:** 2021-02-17

**Authors:** Edwin Valarezo Añazco, Seung Ju Han, Kangil Kim, Patricio Rivera Lopez, Tae-Seong Kim, Sangmin Lee

**Affiliations:** 1 Department of Information Convergence Engineering, Kyung Hee University, Yongin 17104, Korea; edgivala@khu.ac.kr (E.V.A.); sscandidate22@khu.ac.kr (S.J.H.); kimkangil@khu.ac.kr (K.K.); patoalejor@khu.ac.kr (P.R.L.); tskim@khu.ac.kr (T.-S.K.); 2 Faculty of Engineering in Electricity and Computation, FIEC, Escuela Superior Politécnica del Litoral, ESPOL, Guayaquil EC090112, Ecuador

**Keywords:** patchable IMU, six-axis inertial sensor, hand gesture recognition, recurrent neural network, control gestures

## Abstract

Recording human gestures from a wearable sensor produces valuable information to implement control gestures or in healthcare services. The wearable sensor is required to be small and easily worn. Advances in miniaturized sensor and materials research produces patchable inertial measurement units (IMUs). In this paper, a hand gesture recognition system using a single patchable six-axis IMU attached at the wrist via recurrent neural networks (RNN) is presented. The IMU comprises IC-based electronic components on a stretchable, adhesive substrate with serpentine-structured interconnections. The proposed patchable IMU with soft form-factors can be worn in close contact with the human body, comfortably adapting to skin deformations. Thus, signal distortion (i.e., motion artifacts) produced for vibration during the motion is minimized. Also, our patchable IMU has a wireless communication (i.e., Bluetooth) module to continuously send the sensed signals to any processing device. Our hand gesture recognition system was evaluated, attaching the proposed patchable six-axis IMU on the right wrist of five people to recognize three hand gestures using two models based on recurrent neural nets. The RNN-based models are trained and validated using a public database. The preliminary results show that our proposed patchable IMU have potential to continuously monitor people’s motions in remote settings for applications in mobile health, human–computer interaction, and control gestures recognition.

## 1. Introduction

Human motion recognition is a context-aware technology to classify actions or body gestures into labels based on the motion patterns in the data collected from wearable sensors, such as accelerometers and gyroscopes [1]. Technological advances in wireless communication, micro sensor modules, and low power electronic devices make it possible to build highly reliable wearable systems. [2]. The biometric information provided from the wearable devices can be used for activity recognition applications such as health monitoring [3,4,5,6,7], dependency care [8,9], or sports training analysis [10,11,12,13,14,15,16]. Recognizing various hand gestures using inertial measurement units (IMUs) can provide essential contextual information for user control interfaces [17,18,19] or rehabilitation applications [8,9]. Two issues had been raised regarding advanced human motion analysis. First, the sensor and its composition. An IMU built from flexible or stretchable materials allows the sensor to be attached directly to the skin, reducing motion artifact [20]. Second, the human motion recognition algorithm. Recently deep learning has been adopted to create motion recognition systems because it does not need feature extraction and selection [21,22].

Recently, wearable and patchable sensors using flexible/stretchable substrates have been implemented [1,7]. The patchable sensors (i.e., bioelectronics sensors) can adapt to the human body to measure human motions with high fidelity. Typically, commercial wearable IMUs are used with tight straps to avoid mechanical sliding during motion or breathing. However, the bioelectronics sensor with soft form-factors can be in close contact with the human body to minimize the motion artifact [23]. Recent research has focused on implementing highly-scalable and modularized epidermal electronic sensors [24,25], which used rigid integrated circuit (IC) components with robust stretchable interconnections. The patchable sensor enables continuous data logging for ambulatory monitoring through the integration of wireless communication.

Deep learning (DL) is becoming a new trend in the fields of pattern recognition and machine learning, because of its performance in human motion (i.e., gesture and activity) recognition [21,22]. The DL algorithms extract and learn hidden representation (i.e., features) directly from the raw data. In contrast, a careful feature extraction and selection is needed by traditional machine learning algorithms such as naïve Bayes (NB), K-nearest neighbors (KNN), decision tree (DT), and support vector machines (SVM) [26,27]. Among DL algorithms, recurrent neural networks (RNN) are actively used for activity and hand gesture recognition with IMUs because the recurrent connections favorable process sequential data [28,29]. For instance, Ordoñez et al. [30] proposed a hand gesture and activity recognition system based on RNN with long short term memory (LSTM) from a full set of body-worn IMUs. Their results showed that RNN performs better classification between similar gestures than KNN, DT, and SVM. Vu et al. [31] compared the activity recognition performance of LSTM and gate recurrent unit (GRU) from wearable sensors, i.e., accelerometer and gyroscope signals. Their results showed that GRU performs similar to LSTM in activity recognition, despite GRU having a structure less complex than LSTM.

In this paper, a stretchable and patchable six-axis IMU for human motion recognition via RNN is presented. The proposed patchable IMU is implemented using IC components soldered on bonding pads. To resolve the limitations of conventional sensors, the proposed patchable IMU was implemented on the stretchable adhesive material with serpentine-structured interconnections [32,33,34], which can be comfortably attached to any part of human body. The proposed patchable IMU was implemented using a commercialized, low-cost six-axis inertial sensor, a Bluetooth low energy (BLE) microprotocol system-on-a-chip (SoC), a chip antenna, and several electronic components for real-time biometric data acquisition. Our proposed patchable six-axis IMU was tested for hand gesture recognition using two RNN-based models because of the RNN performance in activity recognition with multiple IMUs reported in previous works [21,22,23,24,25,26,27,28,29,30,31]. First, an RNN model based on LSTM units and a second RNN model based on GRU units were devised. The RNN-based models were validated using a public database. Then, the trained models recognize hand gestures from our patchable IMU attached at the right wrist of five subjects. The results show the RNN-based models achieving similar classification accuracy with the public dataset and our sensed dataset. 

The related works, hardware design, and system implementation process will be represented in detail by following sections. In addition, the methodology for data training and data processing strategies using neural networks will be described in detail for user’s hand gesture recognition. Through the experimental results of this paper, it will be shown that the proposed patchable IMU and deep learning algorithm enable real-time recognition of a user’s hand gestures. We believe that expansion of the preliminary results will enable the development and application of a high-performance multi-parameter sensor microsystem. 

## 2. Related Works

Recently, IMUs are turned into a common sensor used in smartphones, smartwatch, and smart bands because it reduces the cost and physical dimensions [35,36,37]. The popularity of smart and wearable devices generated a growing interest in the research community to use IMUs for motion recognition. Chen et al. [38] proposed a bendable sensing system for motion detection. Their system housed an IMU built on a hard printed-circuit board (PCB) into a rubber band to be attached to human limbs. The rubber-case worked for shock-absorption, avoiding sensor damage. Also, the band can easily attach or detach to collect data from more than one location, i.e., wrists or legs. However, motion artifact (i.e., noise) affected the sensed signals because vibrations are produced in the rubber band during the motion. Hand gestures are especially susceptible to motion artifacts because of the force and speed involved in the motions. Attaching the IMU directly to the skin might reduce the sensed motion artifact, but the general methodology to produce a patchable sensor is complex and expensive. Using our methodology, an inexpensive patchable six-axis IMU can be produced. 

Hand gesture recognition via DL is actively investigated because of its potential in applications such as healthcare and human–computer interaction (HCI). Cole et al. [39] proposed a system to recognize smoking gestures from a tri-axis accelerometer built into an apple watch via an artificial neural network. Their system recognized smoking, eating, drinking, and scratching nose gestures, achieving an accuracy of 70%. Valarezo et al. [40] performed smoking recognition using a six-axis IMU housed on a wrist band via RNN with LSTM units. Valarezo’s system used a two steps classification scheme. First, the IMU signals were classified as activities of daily living or hand gestures. Second, the hand gestures were classified as smoking, eating, and drinking gestures, reporting a smoking accuracy of 91.38%. Nasri et al. [41] used hand gesture recognition for an HCI-based system. In their system, a GRU-based algorithm recognized seven hand gestures to control a 3D game, achieving an accuracy of 82.15% with a new user. Rivera et al. [42] presented a gesture recognition system using an IMU placed at dominant wrists and a RNN based on GRU units. Their GRU-based system learned the underlying temporal dependencies within the time series to recognize seven gestures, achieving an average classification accuracy of 84.94%.

These previous works show that a hand gesture recognition system must focus on the following components. First, a patchable six-axis IMU can reduce the noise in the sensed activity signals by reducing the vibration comparing to sensors attached using rubber bands. Second, artificial intelligence should be based on high-performance algorithms, such as DL.

## 3. Methods

### 3.1. Design and Implementation of Patchable IMU 

#### 3.1.1. System Design

**Figure 1:**
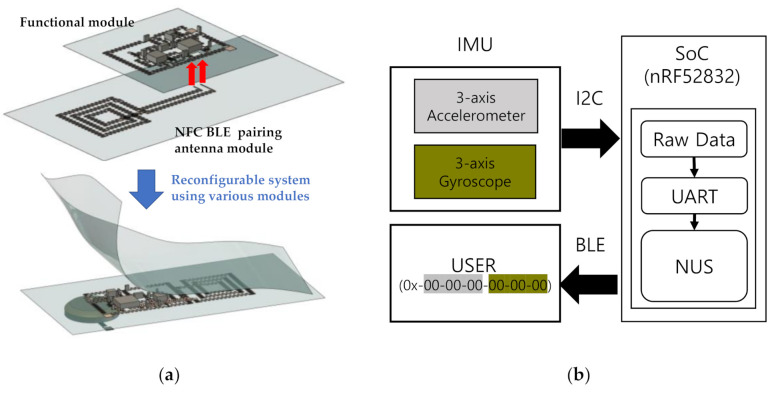
Design of patchable inertial measurement unit (IMU). (**a**) Reconfigurable system concept and schematic; (**b**) system block diagram.

In Figure 1, the schematic and system block diagram of the six-axis patchable IMU is shown. The proposed patchable system consists of an inexpensive, micro six-axis sensor (MPU6050; Invensense, San Jose, CA, USA), low-power BLE SoC (nRF52832; Nordic Semiconductor ASA, Trondheim, Norway), 2.4 GHz chip Blutooth antenna, 32 MHz crystal, and multiple electronic components. All elements used in system configuration are mounted on the bonding footprints, and the bonding footprints are connected by the serpentine-structured interconnections to allow the device to be elongated under various types of mechanical strains. In general, it has been known that human skin can withstand strain of approximately 27% [43]. The top and bottom layers are covered with stretchable adhesive films (Tegaderm; 3M, Saint Paul, MN, USA) to encapsulate the functional components. As is well known, Tegaderm is not a highly-stretchable material and can be permanently deformed when subjected to high levels of stress. However, the reason we used Tegaderm in this system is that the system adheres well to the skin, so it can follow the deformation of the skin precisely. If the material elasticity is too strong, there is a problem that slipping may occur when stretching when attached to the skin, and the Young’s modulus must be similar to that of the skin in order to adapt well to the deformation of the skin. The device is designed with a modular concept that allows components to be reconfigured as needed, enabling the implementation of compact systems optimized for a variety of applications.

The micro six-axis sensor and the SoC transmit inertial signals according to the movement of the attached skin using the two-wired serial interface. In the micro six-axis sensor, three-axes accelerations and three-axes rotational velocities are digitized by the built-in 16-bit analog-to-digital converter (ADC). These converted inertial signals are passed through the bus interface unit of SoC via 400 kHz I2C communication. After the data are processed in the control unit, they are spread by the universal asynchronous receiver/transmitter (UART) structure, and via Bluetooth communication the inertial data are gathered. To reduce noise, especially overshoot in the raw data, a low pass filter is implemented using a damping resistor (33 ohms), and the sampling rate is about 120 Hz. In the case of the MPU-6050, the full-scale of accelerometer and gyroscope can be modified between ±2 g, ±4 g, ±8 g, ±16 g, and ±250 °/s, ±500 °/s, ±1000 °/s, ±2000 °/s, respectively, by converting the code value. When the system is started, the offset is initialized by taking the average of the first 200 input values and subtracting it from the raw data. The offset value is updated every time when the system is started. In this paper, an NFC antenna for BLE pairing is used in addition to the previously reported IMU [23]. The reason for using NFC BLE pairing lies in the convenience of BLE pairing. The pairing proceeds simply by bringing the mobile device for data collection close to the device implemented in this paper, and the transmission of raw data also starts simultaneously. This makes it easier for the user to determine the time the sensor operates when measuring by attaching the device to the human body. In this paper, in order to utilize the NFC A-tag provided by the nRF52832, when designing the footprint, we tried to reduce interference as much as possible by excluding the placement of other elements near the pins of the NFC BLE pairing antenna.

#### 3.1.2. System Implementation Process

**Figure 2:**
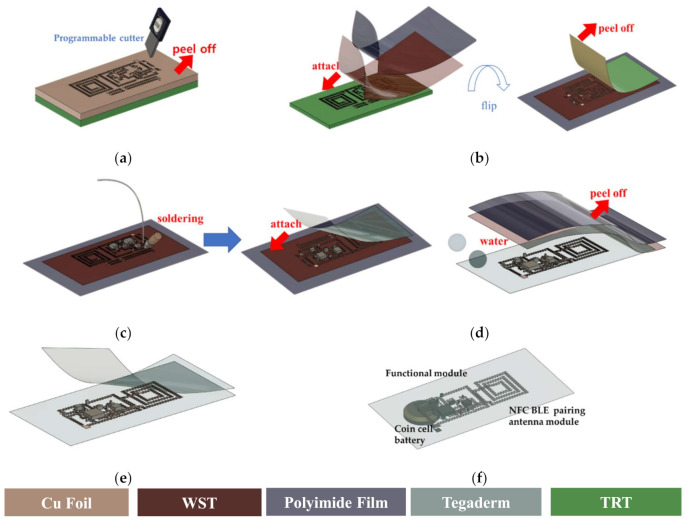
Implementation process patchable IMU. (**a**) Design and formulation of layout footprint; (**b**) water-soluble tape (WST)/polyimide film attachment and thermal release tape (TRT) removal; (**c**) Soldering and top-layer encapsulation; (**d**) Detachment of WST/polyimide film; (**e**) Bottom-layer encapsulation; (**f**) Battery and module alignment.

The implementation procedure of the patchable six-axis IMU on a stretchable adhesive film is described in Figure 2. The footprint of the proposed system is defined by a computer-aided design (CAD) program and programmable cutter (Silhouette Cameo^®^, Silhouette America, Lindon, Utah, USA). This method allows miniaturized devices to be implemented on substrates of various form-factors without the need for expensive cleanroom fabrication processes. The serpentine structure of the metal interconnection is designed to have a line width of 200 μm, and is designed to ensure stable operation even with a tensile strain of up to 30%. The detailed implementation process is as follow. At first, a copper (Cu) foil (Copper 110 Annealed; Online Metals, Seattle, WA, USA) having thickness of 18 μm is laminated on the thermal release tape (TRT). The cutter patterns the TRT-laminated Cu foil according to the pre-programmed CAD footprints. Then, the remaining region, except the designed patterns on TRT-laminated Cu foil, is removed (Figure 2a). Subsequently, a water-soluble tape (WST) and polyimide film are adhered in sequence to the opposite side of the patterned TRT-laminated Cu foil. After bonding on a glass substrate, the whole substrate is heated on a hot plate to peel off the TRT layer as shown in Figure 2b. After the bonding footprint for circuit assembly is revealed, all the components, such as the micro six-axis sensor, signal processing unit, Bluetooth chip antenna, BLE paring antenna, passive elements, and bridge interconnections, are soldered. The whole device is released from the glass substrate after the polyimide film removal. Then, the stretchable adhesive film covers the upper layer of the circuit, as illustrated in Figure 2c, and water drops are used to peel off the WST (Figure 2d). Afterwards, the patchable six-axis IMU is implemented by covering the Tegaderm to the lower part of the electronic components and the serpentine-structured Cu interconnections on a stretchable substrate, as shown in Figure 2e. Finally, alignment of the reconfigurable modules and coin cell battery is performed (Figure 2f).

### 3.2. Hand Gesture Recognition via Recurrent Neural Networks

RNN is an artificial neural network with recurrent connections. The recurrent connections generate a temporal memory, in which the previous state of the network is stored. Then, RNN infers the activity label based on the previous state of the network and the current input data [44]. Unfortunately, RNN might suffer from a vanishing gradient problem with long data sequences [45]. The gradient carries information to update the trainable parameters. In the vanishing gradient problem, the gradient becomes smaller and smaller during the error-backpropagation in training. Then, RNN does not learn because the gradient becomes insignificant. LSTM and GRU units have been developed to overcome learning problems such as the vanishing gradient [46]. An LSTM uses internal paths regulated by gates, where the gradient can flow for long durations [44]. These regulated paths allow error propagation in deep networks.

**Figure 3:**
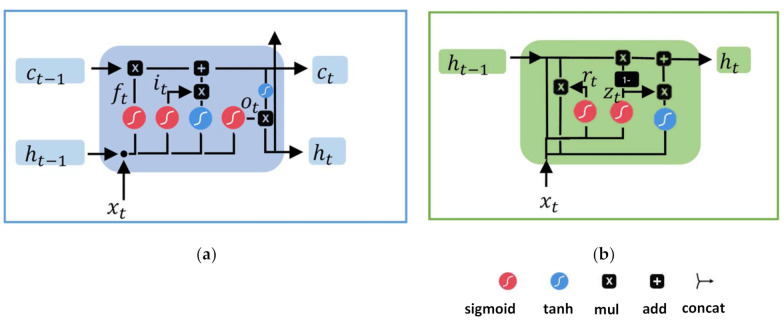
Recurrent units. (**a**) Long short-term memory; (**b**) gate recurrent unit.

Figure 3a shows the structure of the internal gates of an LSTM unit. Equations (1) to (6) describes mathematically the LSTM units, where W is the weights and b the bias.

(1) $$f_{t}=\sigma \left(W\cdot \left[h_{t-1},x_{t}\right]+b\right)$$

(2) $$i_{t}=\sigma \left(W\cdot \left[h_{t-1},x_{t}\;\right]+b\right)$$

(3) $$\tilde{C_{t}}=\tanh\left(W\cdot \left[h_{t-1},x_{t}\right]+b\right)$$

(4) $$C_{t}=f_{t}*C_{t-1}+i_{t}*\tilde{C_{t}}$$

(5) $$o_{t}=\sigma \left(W\cdot \left[h_{t-1},x_{t}\;\right]+b\right)$$

(6) $$h_{t}=o_{t}*\tanh\left(C_{t}\right),$$

GRU is a variant of the LSTM with fewer operations and gates [46]. Figure 3b shows the internal structure of a GRU and Equations (7) to (10) describe the GRU units mathematically.

(7) $$z_{t}=\sigma \left(W_{z}\cdot \left[h_{t-1},x_{t}\right]\right)$$

(8) $$r_{t}=\sigma \left(W_{r}\cdot \left[h_{t-1},x_{t}\right]\right)$$

(9) $${\tilde{h}}_{t}=\tanh\left(W\left[r_{t}*h_{t-1},x_{t}\right]\right)$$

(10) $$h_{t}=\left(1-z_{t}\right)*h_{t-1}+z_{t}*{\tilde{h}}_{t},$$

#### 3.2.1. RNN with Bidirectional LSTM

Bidirectional LSTM (BiLSTM) is a neural network based on LSTM with two kinds of connections [47]. One connection goes forward in time helping to learn from previous representations, as in classic LSTM. The other connection goes backward in time, which helps to learn from future representations. 

**Figure 4:**
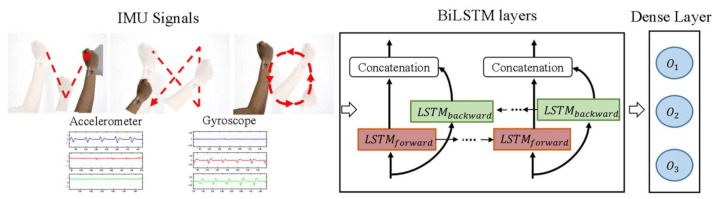
Hand gestures classifier based on a recurrent neural network (RNN) with bidirectional long short term memory (BiLSTM).

Figure 4 shows the hand gestures classifier based on RNN with BiLSTM (i.e., RNN-BiLSTM). From the left, hand gesture signals are sensed using our patchable six-axis IMU. Then, RNN-BiLSTM processes the data using one BiLSTM layer composed of 110 hidden nodes with hyperbolic tangent activation function to extract features. At the output of the network, a dense layer with three nodes and SoftMax activation function provides the classification probability of each class. The dense layer is defined as a fully connected layer. 

Multiclass cross-entropy is the loss function and stochastic gradient descent (SGD) is used to optimize the network parameters. The SGD is described by Equations (11) and (12) where α is learning rate, $W_{t}$ weight matrix, L the loss function, and β the momentum factor set to 0.9.

(11) $$W_{t+1}=\;W_{t}+v_{t+1}$$

(12) $$v_{t+1}=\beta \times v_{t}-\;\alpha \times \nabla L\left(W_{t}\right)$$

#### 3.2.2. RNN with GRU

The GRU is a variant of LSTM, proposed to improve RNN memory with fewer connections than LSTM [46]. GRU only has a reset gate and update gate, while LSTM has cell state, input, forget, and output gates. 

**Figure 5:**
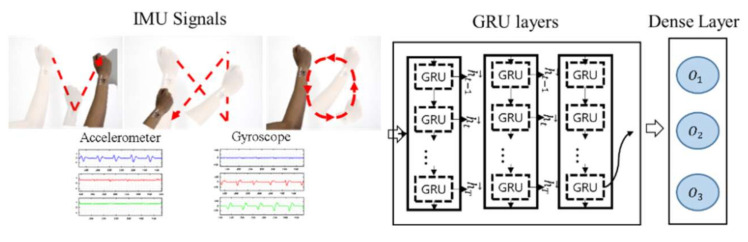
Hand gestures classifier based on RNN with GRU.

Figure 5 shows the hand gesture classifier based on RNN with GRU (i.e., RNN-GRU). The patchable six-axis IMU senses the acceleration and gyroscope signal described during the hand motion. Then, RNN-GRU extracts features from the row IMU signals using three GRU layers with 256, 128, and 64 units, respectively. The output of the last GRU layer goes into a dense layer with three neurons representing each of the hand gestures. Cross-entropy is the loss function and Adam optimizer updates the parameters of the network for training.

### 3.3. RNN-Based Models Implementation

The RNN-BiLSTM was implemented using Deeplearning4J library [48]. The mini-batch approach with a mini-batch size of 100 was used for training. The backpropagation through time (BPTT) backpropagated the gradient. Weight initialization used a random number generator. The learning rate was set as 3 × 10^−2^ and the number of training iterations to 200. The learning rate and the number of training iterations were chosen to avoid overfitting the neural networks to the training dataset. 

The RNN-GRU was implemented using PyTorch library [49]. The GRU-based model trained using mini-batches of 64. Weights were initialized randomly, the learning rate was set to 3 × 10^−4^, and the number of training iterations to 100 steps. 

The RNN-BiLSTM and RNN-GRU classified the data as many-to-one, i.e., after a window of data (3 s) the model infers a single class label. The RNN-based models were trained on a PC with a processor Intel(R) Core(tm) i5-7500 CPU@ 3.40 GHz, 8 GB RAM, and GPU NVIDIA GeForce GTX 1050 Ti.

### 3.4. Database and Data Preparation

The 6DMG public database was used to train the hand gesture recognition models based on RNN. The 6DMG provided information of WorldViz PPT-X4 and Wii Remote Plus (Wiimote) from 21 right-handed and seven left-handed participants, developing 20 hand gestures [50]. The gestures were swipe motions in eight directions, poke motions in four directions, horizontal circles in two directions, vertical circles in two directions, twist roll motion in two directions, and V and X shapes. In our experiment, the four circle motions were merged into a unique circle class. Our training dataset included circle, V, and X shapes because the RNN-based models have an input with fixed time-length. The time-length of the circle, V, and X shapes were similar between them and widely different from the remaining gestures in the 6DMG database. 

The database was preprocessed as follows: the gesture data was down sampled to 50 Hz [51]. The gravity was removed using a fourth order high-pass Butterworth filter with a cutoff frequency of 0.2 Hz [52]. The gestures activity was normalized between 1 to −1 using the maximum and minimum of each channel value. Finally, we put all channels together to create a matrix of 6 by 150. Each matrix corresponds to a single training or testing window.

### 3.5. Evaluation Methodologies

Two evaluation methodologies were used. First, a five-fold test used the data from the 6DMG database. For this test, the data was divided into 80% as a training dataset and the remaining 20% as a testing dataset. Second, we sensed and recognized hand gestures using the proposed patchable six-axis IMU and the RNN-based models. For this second test, a testing dataset was created attaching our patchable IMU at the right wrist of five subjects to perform circle, X, and V shapes, as Figure 4 and Figure 5 show. Each subject performed 50 repetitions of each gesture with a resting time of three minutes after ten repetitions. Our patchable six-axis IMU collected the hand gestures data with a sampling frequency of 50 Hz as is suggested in [51] for gesture and activity recognition. The accelerometer and gyroscope resolution were set to ±16 g and ±2000 °/s, respectively. The collected testing data was preprocessed as the data from the 6DMG public database. 

The classification performance was reported using the accuracy, precision, and recall, which have been widely used for multi-class classification [53]. The performance metrics were computed as,

(13) $$Accuracy=\frac{TP+TN}{TP+TN+FP+FN}$$

(14) $$Precision=\frac{TP}{TP+FP}$$

(15) $$Recall\;=\frac{TP}{TP+FN}$$

where TP is the true positive value, TN the true negative, FP the false positive, FN the false negative.

## 4. Results

### 4.1. Implementation Results of Patchable IMU 

**Figure 6:**
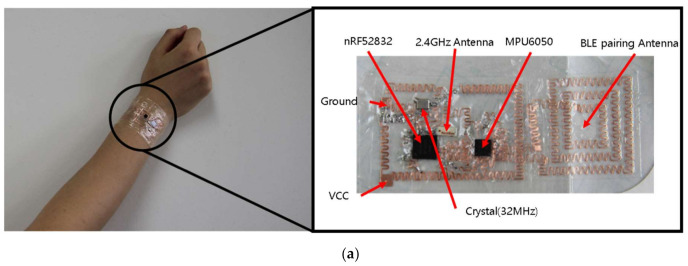

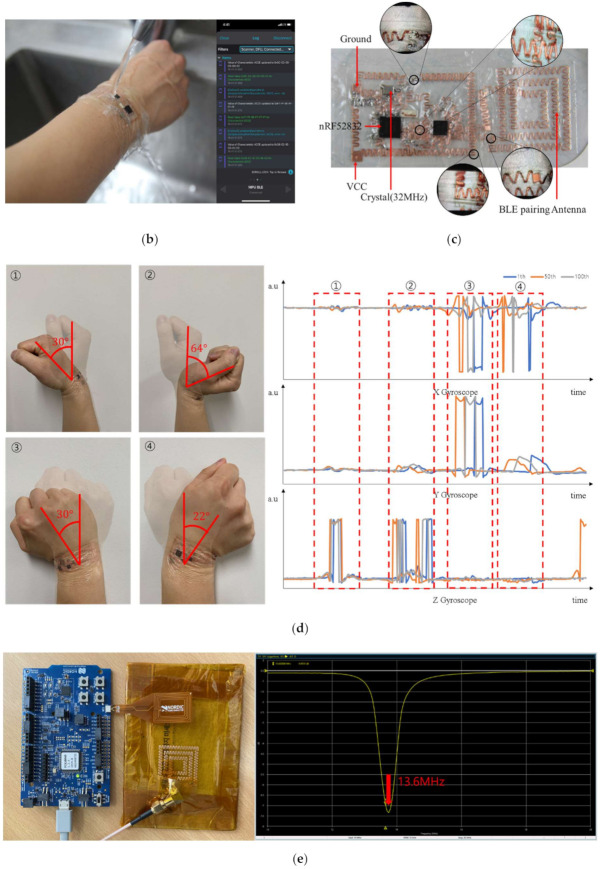
Implementation results. (**a**) Wireless epidermal six-axis IMU; (**b**) waterproof experimental results; (**c**) stretchability test results with 30% tensile strain; (**d**) wrist bending experimental results after first, 50th and 100th repetitions; (**e**) Bluetooth low energy (BLE) paring antenna measurement result in free space.

In Figure 6, the implementation results and characteristic evaluations of the patchable six-axis IMU are presented. As shown in Figure 6a, the overall dimension of the system is 25 mm × 60 mm including the NFC BLE paring antenna, and the thickness is less than 100 μm excluding the ICs and battery. The current consumption is less than 46 mA for full-function operation. In Figure 6b,c, the mechanical evaluation including the stretchability during elongation and waterproof experiment are presented, respectively. Our patchable IMU has a 20 mm × 25 mm, 3-turned conductive coil-type near-field communication (NFC) antenna for BLE paring (line width: 400 μm, line space: 1 mm, half-circle radius: 200 μm and 400 μm, half-circle and half-circle distance: 1 mm). An NFC A-tag is supported on the nRF52832 BLE SoC. An NFC A-tag supports short-range wireless communication that can be used in simplified pairing and payment solutions. Therefore, BLE paring can be performed through an NFC antenna. Figure 6d shows the result after repeated bending after attaching the patchable IMU to the wrist. The left image of Figure 6d shows the bending motion of the wrist used in the experiment in the left, right, upper, and lower directions, and each case has a rotation angle of approximately 30°, 64°, 30°, and 22°, respectively. For each motion case, a total of 10 repeated experiments were conducted for single measurement, and after four different motions, each motion was repeated again to perform a total of 100 measurements. The result on the right in Figure 6d shows the raw signal of the 3-axes gyroscope acquired in the first, 50th and 100th experiments. The experimental results show similar results except for the time axis mismatch over a total of 100 iterations, which shows that the system implemented in this paper is robust against deformation that may occur in repetitive motions.

The verification of NFC BLE paring antenna was conducted by referring to the NRF52832 NFC antenna tuning protocol [32] provided by Nordic Semiconductor with using reference board antenna (NRF52-DK, Nordic semiconductor, Trondheim, Norway) and network analyzer (TTR503A, Tektronix, Beaverton, OR, USA). The result is shown in Figure 6e. The network analyzer uses a custom antenna connected to one port. When the reference board antenna is close (less than 1 cm), the S11 resonant frequency of the network analyzer is close to 13.56 MHz and is adjusted through the capacitor [32]. 

### 4.2. Hand Gesture Classification with Public Database

Table 1 shows the performance of RNN-BiLSTM and RNN-GRU with the public database as a confusion matrix, where the values in the main diagonal are the recall values of each class. The row gestures are the predicted labels and the columns the actual hand gestures. Table 1a shows the classification results of RNN-BiLSTM. Using the public database, RNN-BiLSTM misclassifies 5.15% of the V shape as X shape, 5% of the X shape as circle, and 1.67% of the X shape as V shape gestures. RNN-GRU shows less confusion than RNN-BiLSTM, 0.84% of the V shape as X shape gestures and 1.67% of the X shape is recognized as V shape, as Table 1b shows. The average classification accuracy using RNN-BiLSTM is 96.06% and RNN-GRU 99.16%.

**Figure 7:**
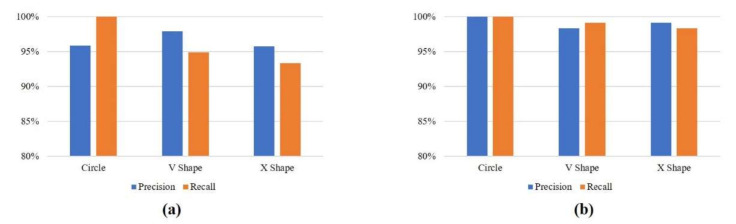
Precision and recall with the public database. (**a**) RNN-BiLSTM; (**b**) RNN-gate recurrent unit (GRU).

Figure 7 shows the precision and recall values of the RNN-based models with the public database. RNN-BiLSTM has a precision of 95.89%, 97.87%, and 95.73% for circle, V shape, and X shape, respectively. The recall values are 100%, 94.85%, and 93.33% for circle, V shape, and X shape, as Figure 7b shows. Figure 7b shows the classification results of RNN-GRU as a precision of 100% for circle, 98.33% for V shape, and 99.16% for X shape; the recall values are 100% for circle, 99.16% for V shape, and 98.30% for X shape.

### 4.3. Hand Gesture Classification with Collected Dataset

Table 2 shows the classification results of RNN-based models with the collected data as the confusion matrix. Table 2a shows the results of RNN-BiLSTM. With the collected data, RNN-BiLSTM showed most of the misclassification between V and X shapes. Table 2b shows the results of RNN-GRU. With the collected data, RNN-GRU misclassified 2.50% of the circles as X shapes, 3.28% of the V shape as X shape gestures, and 8.20% of the X shape as V shape gestures. The average classification accuracy is 94.12% with RNN-BiLSTM and 95.34% with RNN-GRU.

**Figure 8:**
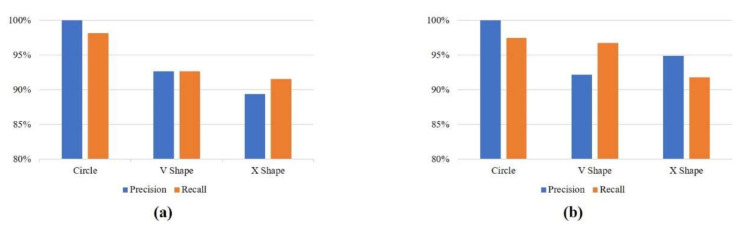
Precision and recall with the collected data. (**a**) RNN-BiLSTM; (**b**) RNN-GRU.

Figure 8a,b show the precision and recall values achieved by RNN-BiLSTM and RNN-GRU with the collected data. RNN-BiLSTM shows precision values of 100% for circles, 92.66% for V shapes, and 89.41% for X shapes. The recall values are 98.15% for circles, 92.66% for V shapes, and 91.57% for X shapes. RNN-GRU has precision values of 100%, 92.19%, and 94.92% for circles, V shapes, and X shapes, respectively. The recall values are 97.50%, 96.72%, and 91.80% for circles, V shapes, and X shapes.

## 5. Discussion

Our patchable IMU was implemented using the serpentine-structured metal interconnections to connect the internal elements. Then, the IMU can stretch and recover according to the motion or rotation of the joint, i.e., right wrist. Also, the inner circuitry can be protected from dust and moisture because of the semipermeable characteristics of the Tegaderm. The BLE continuously transmitted despite water reaching the device.

Table 1 and Table 2 show a slight change in the classification results of the RNN-based models if the testing dataset uses the public or our collected data. The average classification accuracy of RNN-BiLSTM changes from 96.06% to 94.12% using the public and our collected data, respectively. Likewise, using RNN-GRU, the average classification accuracy changed from 99.16% with the public data to 95.34% with our collected data. Then, the hand gesture signals sensed with our patchable six-axis IMU had similar features compared to the signals sensed with a commercial IMU in the Wiimote (i.e., public database).

Analyzing each gesture independently, the circle shape had the highest precision and recall values in all tests with RNN-BiLSTM and RNN-GRU. V and X shapes generated most of the confusion because of the similarity between them. Using RNN-BiLSTM, there was a confusion rate about 5% between V and X shapes with the public database and 8% with the collected data. Using RNN-GRU, about 1% of the V and X shapes we gate recurrent unit (GRU) re misclassified with the public data and 3% to 8% with the collected data. To increase the performance metrics (i.e., accuracy, precision, and recall) with our patchable IMU, the RNN-based models can be fine-tuned with our collected data [21,54].

Future works will focus on extending the experiments of our patchable six-axis IMU to activity monitoring because our patchable IMU can be easily and comfortably attached to all body areas, such as chest or waist. Furthermore, additional experiments should be conducted to analyze the effect of the noise reduction on the classification accuracy, considering our patchable IMU is attached directly to the skin and IMUs are prone to motion artifacts if they are attached to clothes or bands [20,22]. In addition, we are focusing on wireless power charging based on NFC. This study shows preliminary results for BLE pairing by NFC, and we are conducting research to extend the usage time of devices through NFC-based wireless charging in the near future.

## 6. Conclusions

This paper presented a hand gesture recognition system using our designed patchable six-axis IMU via recurrent neural networks. For the patchable IMU, the characteristic evaluations of mechanical properties were performed for applications in ambulatory environment use. The device proposed in this paper was implemented through rigid IC-based circuit elements on a stretchable adhesive film with serpentine-structure interconnections. The size of the implemented system has a length of 25 mm, width of 60 mm, and total thickness of less than 100 μm, excluding the battery and ICs. The results showed the feasibility of using our proposed patchable six-axis IMU for continuously monitoring hand gestures in remote settings via recurrent neural nets. Due to the fact that our six-axis IMU had similar sensing sensitivity to other commonly used IMUs such as the IMU in the Wiimote, a public database could be used to train an RNN-based model for gesture recognition and our six-axis IMU to continuously infer hand gestures labels. The patchable system with soft form-factor introduced in this paper is considered to be low-cost and fully-reconfigurable, enabling its implementation in compact systems for applications in human computer interaction and control gesture recognition in remote settings.

## Figures and Tables

**Table 1:** Hand gestures classification performance with public data. (**a**) RNN-BiLSTM; (**b**) RNN-GRU.

(**a**)			
**(%)**	**Circle**	**V Shape**	**X Shape**
Circle	100	0	0
V Shape	0	94.85	5.15
X Shape	5	1.67	93.33
(**b**)			
**(%)**	**Circle**	**V Shape**	**X Shape**
Circle	100	0	0
V Shape	0	99.16	0.84
X Shape	0	1.67	98.33

**Table 2:** Hand gestures classification performance with collected data. (**a**) RNN-BiLSTM; (**b**) RNN-GRU.

(**a**)			
**(%)**	**Circle**	**V Shape**	**X Shape**
Circle	98.14	0.93	0.93
V Shape	0	92.66	7.34
X Shape	0	8.43	91.57
(**b**)			
**(%)**	**Circle**	**V Shape**	**X Shape**
Circle	97.50	0	2.50
V Shape	0	96.72	3.28
X Shape	0	8.20	91.80
